# QTL mapping for plant height and ear height using bi-parental immortalized heterozygous populations in maize

**DOI:** 10.3389/fpls.2024.1371394

**Published:** 2024-03-25

**Authors:** Haoxiang Yang, Ziran Zhang, Ning Zhang, Ting Li, Junjie Wang, Qing Zhang, Jiquan Xue, Wanchao Zhu, Shutu Xu

**Affiliations:** College of Agronomy, Key Laboratory of Maize Biology and Genetic Breeding in Arid Area of Northwest Region, Yangling, Shaanxi, China

**Keywords:** maize, plant height, ear height, QTL mapping, bi-parental immortalized heterozygous populations

## Abstract

**Introduction:**

Plant height (PH) and ear height (EH) are key plant architectural traits in maize, which will affect the photosynthetic efficiency, high plant density tolerance, suitability for mechanical harvesting

**Methods:**

QTL mapping were conducted for PH and EH using a recombinant inbred line (RIL) population and two corresponding immortalized backcross (IB) populations obtained from crosses between the RIL population and the two parental lines.

**Results:**

A total of 17 and 15 QTL were detected in the RIL and IB populations, respectively. Two QTL, *qPH1-1* (*qEH1-1*) and *qPH1-2* (*qEH1-4*) in the RIL, were simultaneously identified for PH and EH. Combing reported genome-wide association and cloned PH-related genes, co-expression network analyses were constructed, then five candidate genes with high confidence in major QTL were identified including *Zm00001d011117* and *Zm00001d011108*, whose homologs have been confirmed to play a role in determining PH in maize and soybean.

**Discussion:**

QTL mapping used a immortalized backcross population is a new strategy. These identified genes in this study can provide new insights for improving the plant architecture in maize.

## Introduction

Maize (*Zea mays* L.) is one of the world’s most important crops, as it plays a key role in ensuring food security and promoting economic development (Hossain et al., 2022). The “Green Revolution” addressed the problems associated with the excessive plant growth and lodging caused by extensive fertilization through the development of dwarf varieties in crops, such as wheat and rice (Khush, 2001; Evenson and Gollin, 2003; Hedden, 2003). Much progress has been made in our understanding of the plant architecture of crops, and ideal plant architecture traits have been identified for various crops (Jafari et al., 2023).

Maize plants are ideal when they have high photosynthetic efficiency and lodging resistance and the canopy structure is optimized (Pan et al., 2017); these traits can directly or indirectly affect grain yield (Zhou et al., 2016). Recent studies have suggested that improving planting density and the mechanization level in production have become critically important for achieving high and stable maize yields (Assefa et al., 2018; Tao et al., 2019). However, increasing planting density results in changes in the characteristics of maize plant architecture, such as an elongated stem and reduced stem strength, which weakens lodging resistance and decreases yield (Wang et al., 2019; Fei et al., 2022). Exploring the genetic basis of traits associated with plant architecture is thus important for optimizing the architecture of maize plants and thus ensuring that maize plants are adapted to a future of high-density production.

Plant architectural traits, such as plant height (PH) and ear height (EH), are typical polygenic traits in maize that are affected by environmental changes. The use of molecular marker technology to identify quantitative trait loci (QTL) for maize plant architecture traits can shed light on the genetic basis of these traits. Various populations and methods have been used to genetically dissect PH, EH, and other plant architecture traits in maize. Numerous QTL associated with these traits have been identified. Bai et al. utilized maize near-isogenic lines and backcross populations for the QTL mapping of PH and EH, and they detected nine QTL associated with PH and 15 QTL associated with EH, which explained between 3.1% and 31.2% of the phenotypic variation (Bai et al., 2010). Li et al. identified a candidate gene for PH and EH encoding a C2H2 zinc finger family protein (*GRMZM2G114667*) in the shared region using linkage and association mapping (Li et al., 2016). Sa et al. detected three QTL related to PH and five QTL related to EH using a recombinant inbred line (RIL) population, which explained between 3.79% and 19.85% of the phenotypic variation (Sa et al., 2021). Fei et al. identified three candidate genes located in the three consistent QTL regions using two populations, and these genes were involved in the gibberellin (GA)-activated signal pathway, brassinolide signal transduction pathway, and auxin-activated signal pathway (Fei et al., 2022).

Additionally, maize is one of the best crops for heterosis utilization, which required to clear the heterosis using F_1_ hybrids. Researchers have utilized various genetic designs to explore heterosis-related QTL for maize PH and EH. Li et al. Identified 14 QTL for PH and EH using two IB populations and found that overdominance (OD) effects were the major contributors to the heterosis observed in PH and EH (Li et al., 2017). Xiao et al. employed the Complete-diallel design plus Unbalanced Breeding-like Inter-Cross (CUBIC) population to conduct Genome-Wide Association Studies (GWAS) for PH traits and the corresponding mid-parent heterosis (MPH). They detected two significant peaks in maternal populations and F_1_ populations for MPH of PH, and demonstrated the epistatic interactions that Brachytic2 represses the Ubiquitin3 for PH (Xiao et al., 2021).

Several genes affecting PH and EH in maize have been successfully cloned, and most of these genes are associated with hormones such as auxin, GA, and brassinolide. The representative gene *Dwarf1*(*D1*), which was also referred to as *ZmGA3ox2*, affects PH by influencing GA biosynthesis (Phinney, 1956; Spray et al., 1996; Teng et al., 2013). Multani et al. cloned the *ZmBr2* gene, which shortens maize internodes and controls PH and EH (Multani et al., 2003). Subsequently, Xing et al. confirmed that *ZmBr2* altered PH by influencing the polar transport of auxin and reducing the number of longitudinal cells in maize (Xing et al., 2015). Li et al. confirmed that *ZmRPH1* overexpression reduces maize PH and EH, which enhances resistance to lodging (Li et al., 2020). Liu et al. found that *ZmGA2ox3* influences GA biosynthesis, and the loss of function of this gene results in maize dwarfism (Liu et al., 2024).

The main commercially cultivated varieties of maize currently used are single-cross hybrids showing heterosis. The use of hybrid varieties to construct genetic populations might facilitate the identification of loci and genes, as well as characterization of gene function; such studies can also provide valuable insights that could aid the breeding of single-cross hybrids with desirable plant architecture traits.

Here, the commercial hybrid Shaandan 650 (KA105×KB024) and its advanced generation recombinant inbred lines (RILs) were used as experimental materials. Two permanent backcross populations (IB) were constructed by back-crossing RILs with the two parents following the NC III genetic mating design. Using phenotypic data from four different locations, QTL mapping was performed on the two IB populations and the RIL population using GAHP software. Five candidate genes associated with PH and EH were identified using data from multiple public databases. The results of this study can be used to identify the key genes determining plant architecture traits in maize.

## Materials and methods

### Plant materials and field experiment

One RIL population consisting of 183 F_2:9_ lines was developed by crossing inbred lines of KA105 from the Shaan A group with KB024 cultivated from the Shaan B group. Next, the 183 RILs were back-crossed with the two parent lines (KA105 and KB024) based on the NC III design, and two immortalized backcross populations (IB) both contained 183 F_1._ The IB population generated by crossing with KA105 was called IB1, and the IB population generated by crossing with KB024 was called IB2 (Zhang et al., 2022).

The two parent lines, F_1_ (Shaandan650, a nationally approved variety in China), the RIL population, and IB1 and IB2 populations were all planted at four different locations including Sanya (SY, 18.24°N, 109.51°E), Yangling (YaL, 34.28°N, 108.06°E), Yulin (Yul, 34.48°N, 109.58°E), and Weinan (WN, 34.96°N, 109.34°E). This experiment adopts a randomized block design. For each material, planting is carried out in a single row with a length of 4 m. The distance between every two plants within each row is approximately 22 cm, and the distance between every two rows is approximately 0.6 m. The overall density is 75,000 plants/ha row.

### Analysis and collection of phenotypic data

At harvest, phenotypic data, including PH and EH, were collected from 10 representative plants of each material. Basic descriptive analysis of PH and EH of the three populations was conducted across four environments using IBM SPSS Statistics v23 software (https://www.ibm.com/products/spss-statistics). The R package “corrplot” was used to calculate the correlation coefficients between environments and traits (Wei et al., 2017).

Next, we used the R package “lme4” to perform the best linear unbiased prediction (BLUP) to mitigate the effects of environmental factors (Bates et al., 2014). The formula was as follows: Pheno ~ (1|Loc) + (1|Line), where Pheno represents the trait data, Loc refers to all environments, and Line refers to each material (inbred lines or backcross F_1_ hybrids). The parentheses indicate random effects. The model matrix and grouping factors are separated by the vertical bar character “|”. In addition, the broad-sense heritability (*H^2^*) of the traits in RIL, IB1, and IB2 across all locations was estimated using the formula *H^2^ =* $\sigma_{g}^{2}$ */ (* $\sigma_{g}^{2}$ *+* $\sigma_{e}^{2}$ */n)*, where $\sigma_{g}^{2}$ represents the genotypic variance, $\sigma_{e}^{2}$ represents the error variance, and *n* represents the number of environments (Fu and Wang, 2023).

### Genotype detection and genetic linkage map construction

Leaf samples at the five-leaf stage were collected from the 183 RILs and two parental lines (KA105 and KB024) for genomic DNA extraction and analysis. Genomic DNA was extracted using an improved CTAB method (Murray and Thompson, 1980), and the quality of the DNA products was assessed using agarose gel electrophoresis and a UV spectrophotometer. The qualified DNA samples were genotyped using the Maize 6H-60k SNP chip in Beidahuang (Beidahuang Kenfeng Seed Co., Ltd.). The chip was designed using the maize inbred line B73 version 3 (Ref3) genome as a reference by the Maize Research Center of Beijing Academy of Agriculture and Forestry Sciences.

The genotype of the RILs and parents was used to construct the linkage map via the following steps: 1) selection of polymorphic markers in KA105 and KB024; 2) filtering of markers using IciMapping v4.2 (Meng et al., 2015) based on a missing rate ≥ 10% and distortion value P ≥ 0.0001; 3) division of selected effective markers into bins using the “BIN” function; and 4) construction of the genetic linkage map using the “MAP” function with default parameters.

### QTL analysis

QTL analysis was performed separately for PH and EH in the RIL, IB1, and IB2 populations using the inclusive composite interval mapping method and the “QHP” function in GAHP v1.0 software, which was specifically designed to perform QTL mapping in the IB population (Zhang et al., 2022). At the same time, this software allows for QTL combined analysis involving RIL, IB1, and IB2 populations (Zhang et al., 2022). For the sake of convenience in description, we consider this combined analysis as one population and refer to it as the IBL population. In GAHP, QTL identified in the RIL population can be used to estimate additive effect (a). IB1 and IB2 populations can be used to estimate a-d and a+d, respectively. The IBL population can be used to estimate the additive effect (a) and dominance effect (d). Therefore, for QTL identified in the IBL population, the mode of action of the QTL can be determined by calculating the dominance ratio |d/a| (Stuber et al., 1987). The classification criteria are as follows: 1) additive effect (Add): 0< |d/a| ≤ 0.20; 2) partial dominance (PD): 0.20< |d/a|< 0.80; 3) dominance (Dom): 0.80 ≤ |d/a|< 1.20; and 4) overdominance (OD): |d/a| ≥ 1.20 (Jiang et al., 2015).

The LOD value and the related LOD threshold were determined by performing 1,000 permutations, with the Type I error rate set to 0.05 and the PIN set to 0.005. If the detected QTL intervals for the same trait are identical or overlapping, and they exhibit consistent effects, they are considered the same QTL. QTL that were consistently detected for different traits in one given population were considered pleiotropic QTL (Frascaroli et al., 2007). The QTL were named following the standard nomenclature, which involves using the prefix “q” followed by the capitalized abbreviation of the trait, the chromosome number, and the position number (Tanksley and McCouch, 1997). For example, the first QTL for the trait “PH” located on chromosome 1 would be denoted “*qPH1-1*”.

### Filtering and annotation of candidate genes

QTL with high PVE (≥10%), or detected in at least two environments or two traits, were denoted as major-QTL. According to the RefGen_v3 reference genome map from Maize GDB (https://maizegdb.org/), we identified all genes within the major-QTL regions. Subsequently, the obtained gene IDs were converted from the third version (v3) to the fourth version (v4) for the purpose of facilitating subsequent comparisons with the database (https://maizegdb.org/).

Candidate genes were identified using integrated network datasets and genome-wide association study (GWAS) results. After converting the genes within the major-QTL intervals to the maize genome v4, a comprehensive comparison was conducted with genes associated with significantly correlated SNPs related to PH and EH in the GWAS Atlas (https://ngdc.cncb.ac.cn/gwas/) and genes that have been proven to be related to maize PH and EH, along with their interacting genes, in the maize gene integrated network (Feng et al., 2023; Han et al., 2023) (v4 version). Genes present in all three sets were considered as candidate genes. Concurrently, based on the maize integrated network, an interaction network analysis was performed for candidate genes and their interacting genes. Genes were annotated using the following databases: Ensembl Plants (http://plants.ensembl.org/index.html), Maize GDB, GWAS Atlas, and NCBI (https://www.ncbi.nlm.nih.gov/). Finally, Gene Ontology (GO) analysis of the candidate genes was performed using the agriGO web server (http://bioinfo.cau.edu.cn/agriGO/index.php), and Kyoto Encyclopedia of Genes and Genomes (KEGG) pathway enrichment analysis was performed using the Kobas web server (http://kobas.cbi.pku.edu.cn/), with a p-value ≤ 0.01 and false discovery rate (FDR)< 0.05 (Kanehisa et al., 2012; Tian et al., 2017).

## Results

### Phenotypic variation of PH and EH in the RIL and IB populations

**Figure 1:**
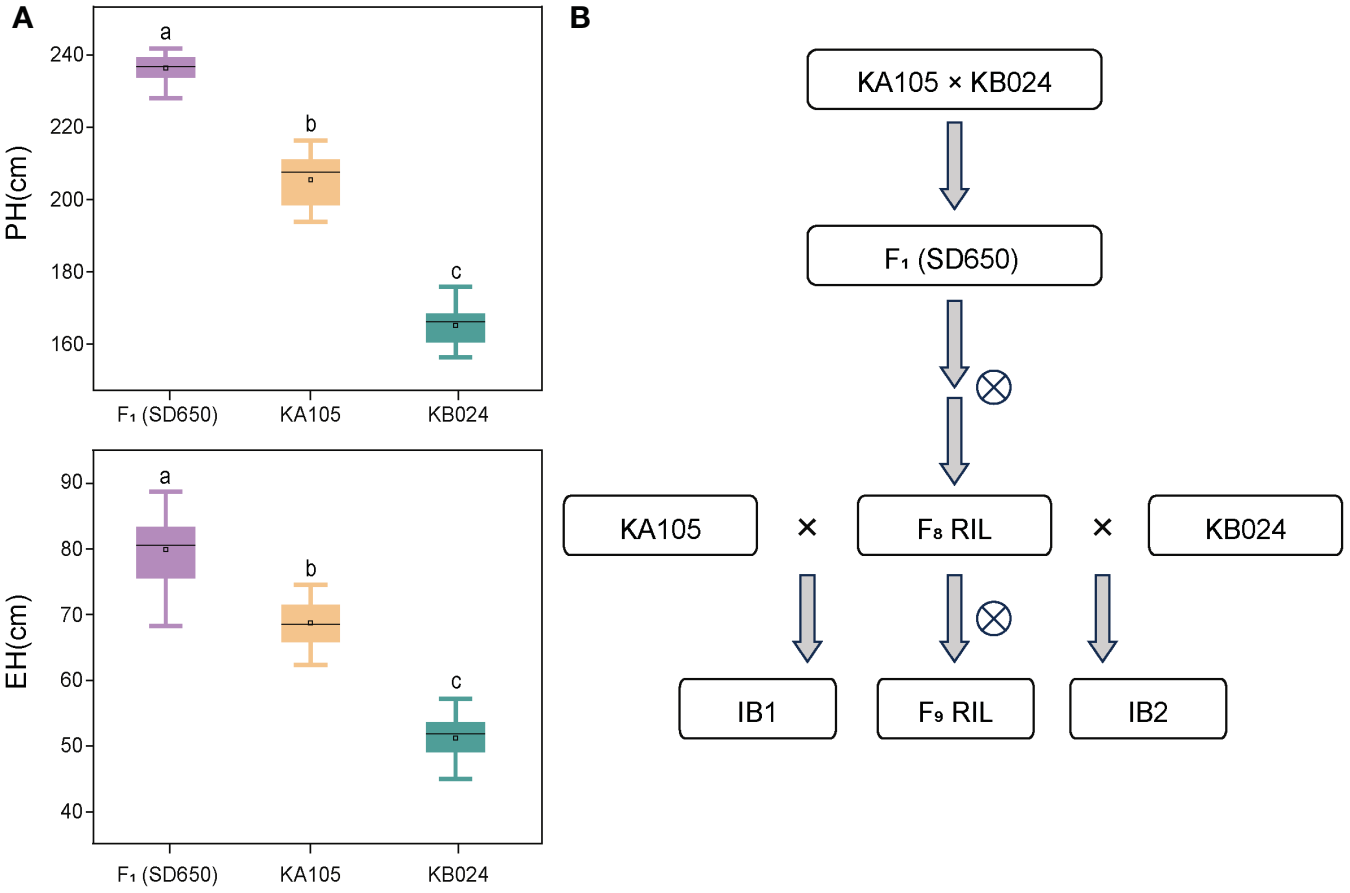
**(A)** represent the PH and EH performance of the hybrid SD650 and its parents KA105 and KB024; a, b, and c represent significant differences at p < 0.001. **(B)** The population construction process. PH, plant height; EH, ear height. ⨂ indicated self-pollinated.

**Figure 2:**
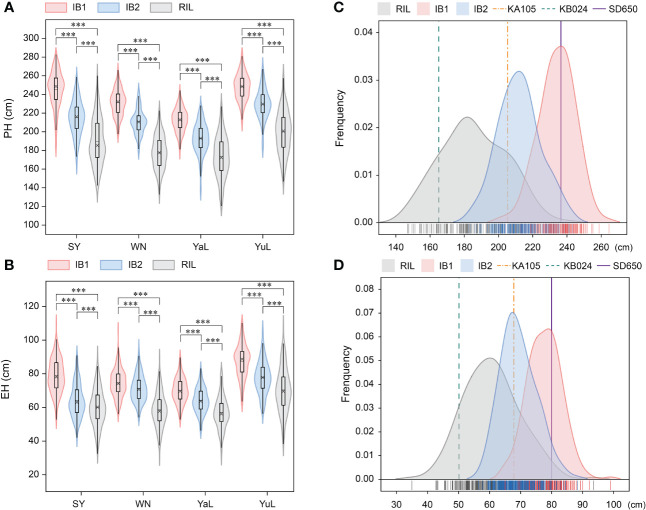
The phenotype variation and distribution of PH and EH in RIL and two IB populations. **(A)** The phenotypic variation of PH in three populations under four locations. **(B)** The phenotypic variation of EH in three populations under four locations. **(C)** The phenotypic distribution of PH BLUP in the RIL, IB1, and IB2 populations. **(D)** The phenotypic distribution of EH BLUP in the RIL, IB1, and IB2 populations. PH, plant height; EH, ear height. *** indicates a significant difference at P < 0.001.

Investigation of the PH and EH of two parents (KA105 and KB024) and their F_1_ progeny at multiple locations revealed significant differences in PH and EH among hybrids (**Figure 1A**). KA105 and KB024 were used to construct the RIL population and corresponding backcross populations (IB1 and IB2) for dissecting the genetic mechanism of PH and EH (**Figure 1B**). Finally, 183 individuals were obtained in each population. All individuals were planted in four locations, and the BLUP of the PH and EH data was used in subsequent analyses. The average PH was 185.56 cm, 234.16 cm, and 212.07 cm for RIL, IB1, and IB2, respectively (**Table 1**, **Figure 2**). The average EH was 60.70 cm, 77.72 cm, and 69.37 cm for RIL, IB1, and IB2, respectively (**Table 1**; **Figure 2**). The absolute values of the skewness and kurtosis of PH and EH in the RIL and two IB populations were all less than 1 (**Table 1**), indicating that PH and EH were normally distributed and typical quantitative traits.

**Table 1:** Description statistics of phenotypic traits for RIL populations and two IB populations under four environments and BLUP.

Trait	Env	KA105	KB024	RIL						IB1						IB2					
		Mean±SD	Mean±SD	Range	Mean±sd	CV(%)	Ske	Kur	H^2^	Range	Mean±sd	CV (%)	Ske	Kur	H2	Range	Mean±sd	CV (%)	Ske	Kur	H^2^
PH	SY	191.90±7.87	158.83±7.17	142.80-259.75	189.73±24.57	12.95	0.32	-0.42	0.94	184.00-282.40	245.53±18.89	7.70	-0.72	0.68	0.85	165.00-258.40	215.08±18.26	8.49	-0.13	-0.15	0.89
	WN	211.50±10.43	149.00±9.22	133.00-222.20	178.25±17.71	9.94	0.05	-0.42		197.80-265.75	231.10±13.82	5.98	-0.08	-0.33		177.67-245.00	210.60±12.99	6.17	0.22	0.12	
	YaL	194.61±10.77	146.48±9.02	120.88-226.90	173.35±21.82	12.59	-0.02	-0.26		177.33-244.00	212.62±12.36	5.81	-0.15	-0.19		137.10-227.71	192.76±16.49	8.56	-0.55	0.96	
	YuL	215.52±7.42	184.23±6.25	147.00-257.10	199.83±22.63	11.33	0.06	-0.34		208.50-288.00	248.14±14.15	5.70	-0.03	-0.04		182.25-269.67	229.64±16.04	6.99	-0.21	0.68	
	BLUP	205.43±6.74	165.07±5.32	146.77-241.10	185.56±19.08	10.28	0.14	-0.50		199.95-264.59	234.16±10.72	4.58	-0.27	0.27		182.30-243.35	212.07±12.30	5.80	0.16	-0.18	
EH	SY	62.89±7.99	48.53±8.37	32.67-91.00	59.96±10.83	18.06	0.12	-0.16	0.90	53.00-110.00	78.88±9.93	12.59	0.09	-0.18	0.80	42.20-90.67	64.20±9.78	15.23	0.45	-0.17	0.82
	WN	67.47±4.39	46.45±7.26	30.60-89.54	58.51±9.97	17.04	0.17	0.13		56.30-106.20	74.57±8.13	10.90	0.38	0.61		54.25-96.30	71.29±7.92	11.11	0.40	-0.11	
	YaL	57.45±5.53	47.33±4.67	30.50-80.33	56.89±9.30	16.35	0.17	0.31		53.00-89.43	70.12±7.33	10.45	0.14	-0.19		46.40-83.00	64.12±7.29	11.38	0.05	-0.20	
	YuL	83.54±8.03	57.32±5.81	38.56-108.00	70.36±12.95	18.41	0.16	-0.15		63.68-110.00	87.47±9.51	10.87	-0.16	-0.07		56.40-106.00	77.92±9.67	12.41	0.23	0.03	
	BLUP	68.74±3.49	51.25±3.76	34.97-93.49	60.70±9.11	15.00	0.31	0.37		64.95-98.96	77.72±5.52	7.10	0.23	0.60		56.42-88.27	69.37±5.78	8.33	0.47	0.04	

In the three populations, transgressive segregation of PH and EH was observed at all four locations and in the BLUP (**Table 1**, **Figure 2A**). In IB1 and IB2, the PH of 97.27% and 90.71% of individuals was significantly higher than that of their parents, respectively. In IB1 and IB2, the EH of 91.80% and 85.25% of individuals was significantly higher than that of their parents, respectively. This indicates a clear hybrid advantage for both PH and EH in the heterozygous populations (IB1 and IB2). Significant correlations in PH and EH between all population were observed (**Supplementary Figure S1**). This suggests that PH and EH may have a similar genetic basis within each population. Furthermore, the PH and EH were higher in IB1 than in IB2 across all four locations, also for those in BLUP; this is consistent with the backcross parents (KA105 and KB024) (**Figure 1B**), suggesting that the hybrid progeny inherited the high PH and EH of KA105. The broad-sense heritability (H^2^) for PH was 0.94 in RIL, 0.85 in IB1, and 0.89 in IB2; the H^2^ for EH was 0.90 in RIL, 0.80 in IB1, and 0.82 in IB2. The H^2^ for both traits was higher in the RIL population than in the two IB populations, and this might be caused by the higher purity of individuals in the RIL than in the IB populations (**Table 1**). In sum, PH and EH in these are typical quantitative traits with high heritability, indicating that genetic factors have a significant effect on PH and EH and thus that they are suitable for QTL analysis.

### Detection of QTL

**Figure 3:**
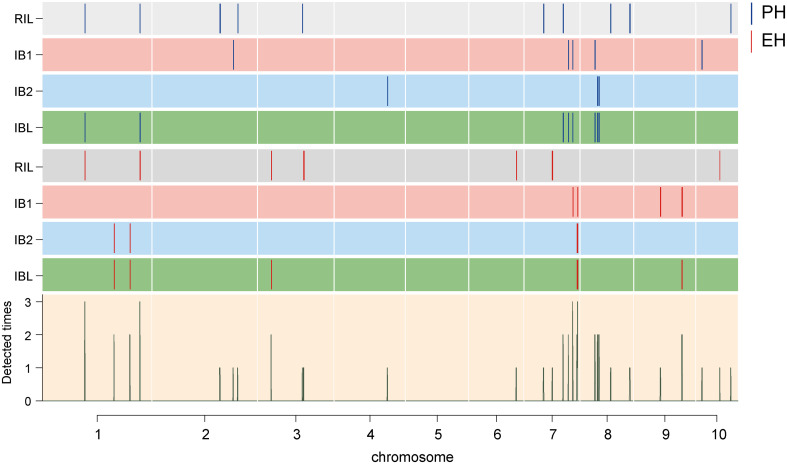
Chromosomal distribution of the identified QTL for PH and EH. The width of the lines shows the length of the confidence interval. The blue and red lines represent the detected PH QTL and EH QTL, respectively. Rectangles of different colors represent different groups. The height of the histogram indicates the frequency of the loci.

**Figure 4:**
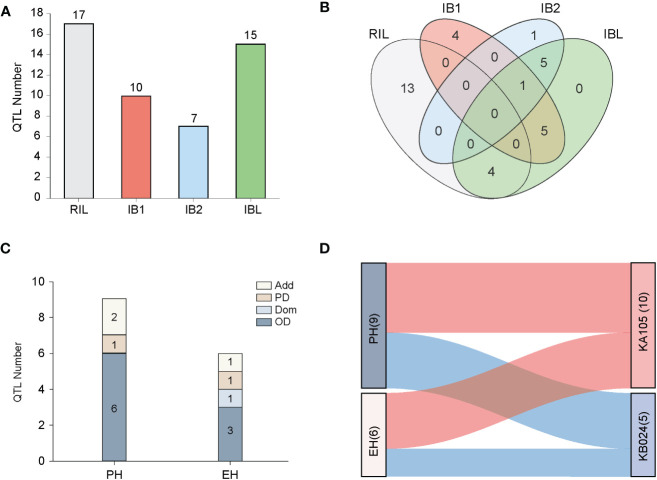
QTL localization and effect analysis. **(A)** The bar chart represents the number of QTL localized in the four populations; **(B)** The overlap of QTL localized in the four populations using Venn diagrams, with different colors indicating different populations. **(C)** The bar chart shows the modes of action of QTL affecting PH and EH traits in the IBL population; **(D)** Indicates the effect sources of QTL affecting PH and EH localized in the RIL and IBL populations, with red indicating effects from the parent KA105 and blue indicating effects from the parent KB024.

Filtering of the SNP markers from the 6H60K SNP chip via IciMapping v4.2 yielded a total of 4,555 high-quality SNPs for the construction of the linkage map. These SNPs were evenly distributed across 10 chromosomes (**Supplementary Figure S2**) and used to construct the linkage map with a total length of 4640.53 cM; the average distance between every pair of markers was 1.04 cM (**Supplementary Table S1**). Next, GAHP v1.0 was used to perform QTL mapping of PH and EH in the RIL and two IB populations. QTL mapping was performed for the RIL, IB1, and IB2 and the integrated IBL population. We identified a total of 49 QTL affecting PH and EH; they were distributed across all 10 chromosomes, and the phenotypic variation explained (PVE) was 0.23–37.26% (PVE) (**Figures 3**, **4A**; **Table 2**). All 15 QTL identified in the IBL can be identified from the RIL or IB populations, and five QTL were co-located in the RIL and IBL, four QTL were co-located in the IB1 and IBL, five QTL were co-located in the IBL and IB2, and one QTL was co-located in the IBL, IB1, and IB2 (**Figure 4B**). In addition, two QTL simultaneously regulated PH and EH in maize, including *qPH1-1*/*qEH1-1*, *and qPH1-2*/*qEH1-4* (**Table 2**).

**Table 2:** The detail information about the QTLs identified in the four populations.

Name	Pop	Chr	Trait	Interval	LOD	LODA	LODD	PVE	PVEA	PVED	add	add-dom	add+dom	dom	|d/a|	Mode
*qPH1-1*	RIL	1	PH	106.55-147.17	24.25	–	–	19.23	–	–	-10.84	–	–	–	–	–
*qPH1-2*	RIL	1	PH	275.98-279.44	16.50	–	–	10.26	–	–	8.00	–	–	–	–	–
*qPH2-1*	RIL	2	PH	189.18-189.18	6.73	–	–	3.69	–	–	-4.80	–	–	–	–	–
*qPH2-3*	RIL	2	PH	213.88-214.05	4.02	–	–	2.11	–	–	3.62	–	–	–	–	–
*qPH3*	RIL	3	PH	141.31-149.28	7.48	–	–	4.08	–	–	-5.02	–	–	–	–	–
*qPH7-1*	RIL	7	PH	24.83-25.83	4.29	–	–	2.31	–	–	3.85	–	–	–	–	–
*qPH7-2*	RIL	7	PH	160.18-161.47	10.62	–	–	6.13	–	–	6.16	–	–	–	–	–
*qPH8-5*	RIL	8	PH	170.42-170.96	5.40	–	–	2.94	–	–	-4.24	–	–	–	–	–
*qPH8-6*	RIL	8	PH	127.66-137.30	3.79	–	–	2.00	–	–	-3.53	–	–	–	–	–
*qPH10-2*	RIL	10	PH	144.63-144.91	5.51	–	–	2.90	–	–	4.23	–	–	–	–	–
*qEH1-1*	RIL	1	EH	106.55-147.17	6.19	–	–	1.04	–	–	-2.76	–	–	–	–	–
*qEH1-4*	RIL	1	EH	275.98-279.44	4.17	–	–	0.62	–	–	2.15	–	–	–	–	–
*qEH3-1*	RIL	3	EH	43.62-45.24	80.27	–	–	37.26	–	–	16.49	–	–	–	–	–
*qEH3-2*	RIL	3	EH	149.83-149.83	5.13	–	–	0.78	–	–	-2.39	–	–	–	–	–
*qEH6*	RIL	6	EH	161.33-162.22	4.46	–	–	0.67	–	–	2.25	–	–	–	–	–
*qEH7-1*	RIL	7	EH	138.72-138.92	3.90	–	–	0.59	–	–	2.11	–	–	–	–	–
*qEH10*	RIL	10	EH	136.96-137.28	5.75	–	–	0.87	–	–	2.53	–	–	–	–	–
*qPH2-2*	IB1	2	PH	207.81-208.02	3.74	–	–	0.96	–	–	–	-5.32	–	–	–	–
*qPH7-3*	IB1	7	PH	167.26-167.26	14.57	–	–	4.26	–	–	–	-11.43	–	–	–	–
*qPH7-4*	IB1	7	PH	168.13-168.44	7.24	–	–	1.93	–	–	–	7.58	–	–	–	–
*qPH8-1*	IB1	8	PH	22.51-22.56	35.08	–	–	13.77	–	–	–	20.25	–	–	–	–
*qPH8-2*	IB1	8	PH	23.89-24.13	24.40	–	–	8.54	–	–	–	-15.87	–	–	–	
*qPH10-1*	IB1	10	PH	6.87-8.15	3.96	–	–	1.06	–	–	–	5.58	–	–	–	–
*qEH7-2*	IB1	7	EH	167.77-167.88	4.55	–	–	4.66	–	–	–	-2.93	–	–	–	–
*qEH7-4*	IB1	7	EH	174.54-174.58	6.24	–	–	6.61	–	–	–	3.48	–	–	–	–
*qEH9-1*	IB1	9	EH	11.46-12.72	3.78	–	–	3.89	–	–	–	-2.67	–	–	–	–
*qEH9-2*	IB1	9	EH	141.03-142.46	7.87	–	–	8.83	–	–	–	4.03	–	–	–	–
*qPH4*	IB2	4	PH	183.24-183.76	3.69	–	–	4.21	–	–	–	–	6.55	–	–	–
*qPH8-3*	IB2	8	PH	116.55-117.35	4.12	–	–	4.81	–	–	–	–	6.87	–	–	–
*qPH8-4*	IB2	8	PH	124.65-128.59	7.87	–	–	9.55	–	–	–	–	-9.75	–	–	–
*qEH1-2*	IB2	1	EH	224.14-225.40	6.31	–	–	1.89	–	–	–	–	3.83	–	–	–
*qEH1-3*	IB2	1	EH	256.73-257.64	3.94	–	–	1.15	–	–	–	–	-2.99	–	–	–
*qEH7-3*	IB2	7	EH	173.29-174.01	19.77	–	–	7.11	–	–	–	–	-7.45	–	–	–
*qEH7-4*	IB2	7	EH	174.54-174.58	26.97	–	–	10.59	–	–	–	–	9.09	–	–	–
*qPH1-1*	IBL	1	PH	106.55-147.17	12.11	12.10	0.01	3.70	3.70	0.00	-5.45	–	–	0.15	0.028	A
*qPH1-2*	IBL	1	PH	275.98-279.44	8.39	8.29	0.10	2.09	2.08	0.01	4.32	–	–	-0.57	0.131	A
*qPH7-2*	IBL	7	PH	160.18-161.47	5.84	5.57	0.27	1.31	1.30	0.02	3.90	–	–	0.96	0.245	PD
*qPH7-3*	IBL	7	PH	167.26-167.26	9.82	3.49	6.33	1.24	0.85	0.38	-3.55	–	–	4.86	1.369	OD
*qPH7-4*	IBL	7	PH	168.13-168.44	5.62	1.34	4.28	0.56	0.31	0.26	1.32	–	–	-3.93	2.984	OD
*qPH8-1*	IBL	8	PH	22.51-22.56	29.34	7.97	21.37	3.80	2.19	1.61	5.46	–	–	-9.83	1.801	OD
*qPH8-2*	IBL	8	PH	23.89-24.13	19.33	5.11	14.22	2.31	1.32	0.99	-3.96	–	–	7.67	1.937	OD
*qPH8-3*	IBL	8	PH	116.55-117.35	4.41	1.02	3.39	0.43	0.23	0.20	1.87	–	–	3.45	1.846	OD
*qPH8-4*	IBL	8	PH	124.65-128.59	8.38	1.81	6.57	0.80	0.41	0.39	-1.84	–	–	-4.86	2.647	OD
*qEH1-2*	IBL	1	EH	224.14-225.40	5.22	2.07	3.15	0.57	0.52	0.05	1.29	–	–	1.42	1.103	D
*qEH1-3*	IBL	1	EH	256.73-257.64	4.84	0.64	4.20	0.23	0.15	0.07	-0.69	–	–	-1.65	2.399	OD
*qEH3-1*	IBL	3	EH	43.62-45.24	41.36	40.00	1.37	2.63	2.60	0.02	8.25	–	–	0.92	0.112	A
*qEH7-3*	IBL	7	EH	173.29-174.01	21.37	5.18	16.19	1.70	1.38	0.32	-1.74	–	–	-3.48	2.005	OD
*qEH7-4*	IBL	7	EH	174.54-174.58	22.23	11.29	10.93	3.78	3.58	0.21	3.52	–	–	2.80	0.796	PD
*qEH9-2*	IBL	9	EH	141.03-142.46	7.00	1.44	5.56	0.45	0.35	0.10	1.05	–	–	-1.92	1.834	OD

In the RIL population, we identified 10 PH QTL and seven EH QTL, which had PVE values ranging from 2.00% to 19.23% and from 0.59% to 37.26%, respectively. In the IB1 population, six PH QTL and four EH QTL were identified, with PVE ranging from 0.96% to 13.77% and from 3.89% to 8.83%, respectively. In the IB2 population, three PH QTL with PVE values of 4.21–9.55% were identified, and four EH QTL with 1.15–10.59% PVE were detected. A total of nine PH QTL and six EH QTL were detected in the IBL population, and the PVE ranged from 0.43% to 3.80% and from 0.23% to 3.78%, respectively (**Table 2**).

In the IBL population, we can detect additive and dominant effects, as well as explore the genetic effects and mode of action for each QTL. The PVEA for QTL mapped in the IBL population was higher than the PVED (**Supplementary Table S2**). However, the absolute values of the add-effects and dom-effects were not consistent with this trend. In some specific QTL, the absolute values of dom-effects were greater than those of add-effects, such as *qPH7-3*, *qPH7-4*, *qPH8-1*, *qPH8-2*, *qPH8-3*, *qPH8-4*, *qEH1-2*, *qEH1-3*, *qEH7-3*, and *qEH9-2*. To better understand the mode of action, we calculated the |d/a| for these 15 QTL from the IBL population and found that 60.00% (nine) of the QTL exhibited OD effects (**Figure 4C**). For PH, six QTL exhibited OD effects, one exhibited a PD effect, and two exhibited an Add effect. For EH, three QTL exhibited OD, one exhibited a Dom effect, one exhibited PD, and one exhibited an Add effect. These results suggested that OD is the main mode of action underlying heterosis for PH and EH in our populations (**Figure 4C**). Consistent with the bi-parental phenotype, the favorable allele of the most identified QTL was inherited from KA105, and the PH and EH were higher in KA105 than in KB024, respectively (**Figure 4D**).

### Identification of candidate genes for PH and EH

**Figure 5:**
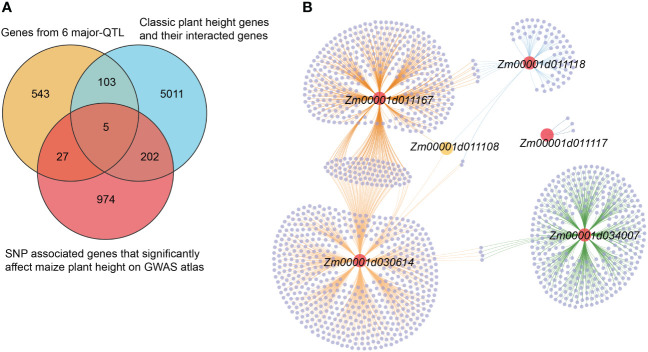
Identification and network of candidate gene. **(A)** Venn diagram of candidate genes for plant architecture within the six major-QTL regions by multiple database. **(B)** The network of five nominated key candidate genes; red dots represent the five key candidate genes selected through comprehensive screening, purple dots represent genes associated with the screened genes, and yellow dot represents the common interacted genes with the three key candidate genes.

QTL with high PVE (≥10%), or detected in at least two environments or for two traits, were designated as major-QTL. Finally, six major-QTL were retained for further analysis, including *qPH1-1* (*qEH1-1*), *qPH1-2* (*qEH1-4*), *qPH7-2*, *qPH8-6*, *qEH3-1*, and *qEH9-2*. All genes within these major-QTL intervals were extracted for comparison with the reported genes identified by GWAS (https://ngdc.cncb.ac.cn/gwas/) (Liu et al., 2023b). Classic PH and EH genes and their interacting genes were obtained from the maize integration network (Feng et al., 2023; Han et al., 2023). Five shared genes were identified, including *Zm00001d030614*, *Zm00001d034007*, *Zm00001d011117*, *Zm00001d011118*, and *Zm00001d011167* (**Figure 5A**). In addition, *Zm00001d011108* near *qPH8-6* interacted with *Zm00001d030614*, *Zm00001d011167*, and *Zm00001d011118* in the network (**Figure 5B**; **Table 3**). Therefore, we designated these six genes as candidate genes that potentially affect plant architecture.

**Table 3:** Information for the six genes screened using the three datasets.

The Gene ID of the Selected Genes (v4)	Source	Gene ID (v3)	Chrom	Gene	Portein Information
*Zm00001d030614*	*qPH1-1*	*GRMZM2G065205*	1	mcm1	DNA replication licensing factor *MCM7*/ replication licensing factor *MCM7*-like protein
*Zm00001d034007*	*qPH1-2*	*GRMZM2G011373*	1	–	–
*Zm00001d011117*	*qPH8-6*	*GRMZM2G363429*	8	–	cytochrome *P450* family 722 subfamily A polypeptide 1
*Zm00001d011118*	*qPH8-6*	*AC199039.3_FG003*	8	–	AC199039.3_FGT003 / Os12g0638500-like protein
*Zm00001d011167*	*qPH8-6*	*GRMZM2G064675*	8	cl26374_1	cl26374_1(629)
*Zm00001d011108*	interaction network	*GRMZM2G339151*	8	vim102	variant in methylation102

**Figure 6:**
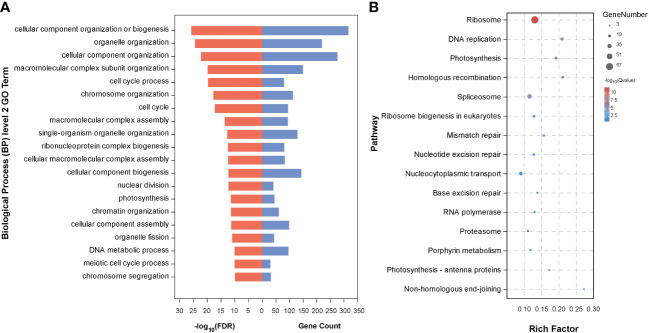
Enrichment analysis of the genes from the interacted genes in **Figure 5**. **(A)** The top 20 secondary GO terms enriched (arranged by -log_10_(FDR)); **(B)** Fourteen enriched pathways by KEGG analysis.

To further determine the biological functions of the five key candidate genes and interacting genes (1,415 genes) in the network, we conducted GO analysis of these genes and found that they were enriched in 211 GO terms, including 113 in Biological Process, 70 in Cellular Component, and 28 in Molecular Function (**Supplementary Table S2**). The top 20 GO terms were mainly related to cell proliferation and cell division, including cellular component organization or biogenesis, organelle organization, cellular component organization, macromolecular complex subunit organization, cell cycle process, and cell cycle (**Figure 6A**). We also performed KEGG pathway analysis and identified 15 significantly enriched pathways, including DNA replication, photosynthesis, homologous recombination, spliceosome, ribosome biogenesis in eukaryotes, mismatch repair, nucleotide excision repair, nucleocytoplasmic transport, base excision repair, and RNA polymerase (**Figure 6B**).

## Discussion

### The advantages of GAHP in QTL detection for bi-parental immortalized heterozygous populations

Genetic dissections of complex quantitative traits have been conducted in an increasing number of populations and species, and this has been driven by the improvement of algorithms and models. In maize, the new representative genetic populations include multi-parent advanced generation inter-cross (MAGIC), complete-diallel plus unbalanced breeding-derived inter-cross (CUBIC), F_1_, and testcross populations. Dell et al. identified the genetic basis of complex agronomic traits using MAGIC populations derived from eight genetically diverse parents (Dell Acqua et al., 2015). Zhou et al. performed QTL mapping for height-related traits and their corresponding general combining ability (GCA) and specific combining ability (SCA) effects using two testcross populations (Zhou et al., 2018). Liu et al. found that epistasis contributes to phenotypic variance in 23 agronomic traits in a CUBIC population descended from 24 elite inbred lines (Liu et al., 2020). Dong et al. conducted genome-wide association mapping of kernel moisture and kernel dehydration using 442 F_1_s derived from 113 inbred lines (Dong et al., 2023). Liu et al. (2023) dissected the genetic basis of nitrogen use efficiency using maize inbred lines and test crosses (Liu et al., 2023a). The results of these studies suggest that genetic analyses of cross populations are effective for clarifying the mode of action of genes in F_1_ populations, which are the most commonly used for commercial varieties.

We performed QTL mapping of plant architecture using three populations: RIL populations from two inbred lines, an immortalized backcross population with the first parent (IB1), and an immortalized backcross population with the second parent (IB2). We identified 15 QTL in the IBL population (the integrated population) using GAHP (https://isbreeding.caas.cn/rj/GAHP/ec4ff2474369420b893f2440696fbff7.htm), which is a new QTL mapping software that can simulate bi-parental immortalized heterozygous and pure-line populations and perform QTL mapping power analysis (Zhang et al., 2022); we further found that all these QTL could be detected in a single population, either RIL or IB1/IB2 (**Figure 3**, **Table 2**). The QTL identified from IBL can explain less phenotypic variation than the corresponding QTL in the single population; they thus provide a more accurate reflection of the effect of each QTL in the hybrids, which will aid subsequent breeding efforts. Furthermore, QTL mapping of the IBL population using GAHP can be used to calculate Add or Dom effects, along with the contributions of Add or Dom to PVE (**Table 2**). All commercial variants of maize, including F_1_ populations, are single hybrids; thus, genetic analysis of heterozygous populations can provide valuable information for evaluating the mode of action of each QTL, and this information can be used to enhance germplasm resources.

### PH and EH show high environmental sensitivity and have a complex genetic basis in maize

Increases in yield observed in previous years have mainly been achieved via increases in plant density (Duvick, 2005), and this can be significantly affected by plant architecture. Therefore, understanding the genetic basis of PH and EH is important for increasing plant density, as well as grain yield. In this study, PH and EH of the RIL, IB1, and IBL populations were investigated across four locations (SY, WN, YaL, and YuL). As expected, the average PH and EH were relatively higher in the two IB populations than in the RIL population due to heterosis, and the average PH and EH were higher in IB1 than in IB2; this is consistent with the higher PH and EH of the test lines (KA105) of IB1 than in the test lines (KB024) of IB2 (**Figures 1A**, **2**). We also found that variation in PH and EH was greater in RIL than in IB1 and IB2, and this stems from the fact that most of the genotypes were heterozygous in each line from IB populations but not in RILs. However, given the higher purity of genotypes in the RIL, the H^2^ values of PH and EH were higher in the RIL than in the two IB populations (**Table 1**). This indicates that the genetic background and phenotypes of the parents play a key role in determining the extent to which plant architecture can be improved via breeding (**Figure 2**).

Genetic analysis of PH and EH in the RIL, IB1, IB2, and the combined population IBL revealed 47 QTL for PH and EH distributed in 29 genomic regions across nine chromosomes, excluding chromosome 5 (**Figure 3**; **Table 2**). Some QTL clustered together, such as *qPH8-1* and *qPH8-2*, which were separated by 1.33 Mb, and *qEH7-3* and *qEH7-4*, which were separated by 0.54 Mb. QTL mapping revealed that *qPH1-1* was co-localized with *qEH1-1* and *qPH1-2* was co-localized with *qEH1-4*, indicating that PH and EH had a partially shared genetic basis; this might explain the significant positive correlations (r ranging from 0.59 to 0.71) between PH and EH. Furthermore, QTL mapping in IBL revealed that PD and OD effects contributed to variation in PH and EH (**Table 2**); this increases the difficulty of dissecting the genetic basis of PH and EH in maize. We did not analyze epistatic interactions in this study, yet these played a role in regulating PH and EH. Although several key genes regulating PH have been cloned, such as *D1* (Teng et al., 2013) and *BR2* (Xing et al., 2015), the regulatory mechanism of PH remains unclear.

### Candidate genes associated with development were identified in this study

Comparison of the reported genes associated with maize PH and EH revealed that the classic gene *Zm00001d041957* (*ZmCCS52B*, regulating maize PH) identified by Yang et al. (2015) is located within the interval of *qPH3* derived from the RIL population in this study, which explained 4.08% of the phenotypic variation. *ZmCCS52B* is a cell cycle switch protein that affects maize PH through its effects on cell division (Yang, 2015). The gene *Zm00001D008909* (*GA2ox9*), which belongs to the GA 2-oxidase (*GA2ox*) family, is located within the interval of *qPH8-2* derived from the IB1 population and IBL population, which explains 8.54% and 2.31% of the phenotypic variation, respectively. The homologous gene *ZmGA2ox3* has been shown to affect PH in maize (Liu et al., 2024), and the proteins encoded by the homologous genes in this family can deactivate endogenous bioactive GA to regulate plant growth. Another gene *Zm0001d010987* (*rap2.7*), which is located within the interval of *qPH8-6* derived from the RIL population with 2.00% PVE, was reported to control maize flowering time. *Zmrap2.7* is an AP2 transcription factor that acts as a flowering suppressor in maize (Salvi et al., 2007; Dong et al., 2012).

Using data from the GWAS Atlas, we found that some significantly associated SNPs affecting PH and EH were co-localized with the QTL identified in this study (**Supplementary Table S3**), suggesting that the reliability of the QTL results was high. Five genes that could be shared in our QTL were identified using information derived from a previously constructed gene regulatory network (Feng et al., 2023; Han et al., 2023) (**Figure 5A**). One gene that interacts with three of these five candidate genes was also identified (**Figure 5A**). *Zm00001d011117* encodes a putative cytochrome P450 superfamily protein, and the homologous gene *ZmD1* has been reported to regulate maize plant architecture (Le et al., 2022). Additionally, *Zm00001d011108* encodes E3 ubiquitin-protein ligase ORTHRUS 2. The E3 ligase encoded by *GmlPA1* has been confirmed to play a role in regulating the PH of soybean (Sun et al., 2023). These findings demonstrate the utility of QTL mapping for complex quantitative traits using multiple populations with diverse genetic backgrounds, and the related candidate genes can be identified rapidly using previously published multi-omic datasets.

## Conclusion

We conducted a genetic analysis of two plant architecture-related traits (PH and EH) in a bi-parental immortalized heterozygous population and identified 34 QTL; most of the QTL effects were derived from the KA105 parent. The QTL, GWAS, and co-expression network revealed six candidate genes (*Zm00001d030614*, *Zm00001d034007*, *Zm00001d011108*, *Zm00001d011117*, *Zm00001d011118*, and *Zm00001d011167*), and the homologous genes of *Zm00001d011117* and *Zm00001d011108* were functionally validated in maize and soybean, respectively. In sum, we identified candidate genes affecting PH and EH via QTL mapping using multiple populations. The results of this study provide new insights that could be used to improve maize plant architecture.

## Data availability statement

The datasets presented in this study can be found in online repositories. The names of the repository/repositories and accession number(s) can be found below: https://ngdc.cncb.ac.cn/. The accession numbers are PRJCA022833 and OMIX005692.

## Author contributions

HY: Data curation, Formal analysis, Writing – original draft, Investigation, Software. ZZ: Investigation, Writing – original draft, Data curation, Formal analysis. NZ: Investigation, Writing – original draft, Data curation, Formal analysis. TL: Data curation, Investigation, Methodology, Software, Writing – original draft, Visualization, Formal analysis. JW: Investigation, Writing – original draft, Data curation, Formal analysis. QZ: Writing – original draft. JX: Resources, Supervision, Writing – review & editing, Project administration. WZ: Methodology, Supervision, Writing – review & editing, Project administration, Visualization. SX: Funding acquisition, Methodology, Supervision, Writing – review & editing, Resources, Visualization.

## Supplementary material

Supplementary Figure 1 Correlation between PH and EH in RIL and two IB populations. The blue bars represent the corresponding phenotype distribution, the red curve is the phenotype distribution curve, and the green curve is the correlation fitting curve for the two phenotype data. *** indicates p<0.001, ** indicates p<0.01, and * indicates p<0.05.

Supplementary Figure 2 Genetic linkage map marker density.
