# Supplementary figures and images for: QTL mapping for plant height and ear height using bi-parental immortalized heterozygous populations in maize
Source: Front Plant Sci. 2024 Mar 25;15:1371394. doi: 10.3389/fpls.2024.1371394 (PMC10999566; doi:10.3389/fpls.2024.1371394)

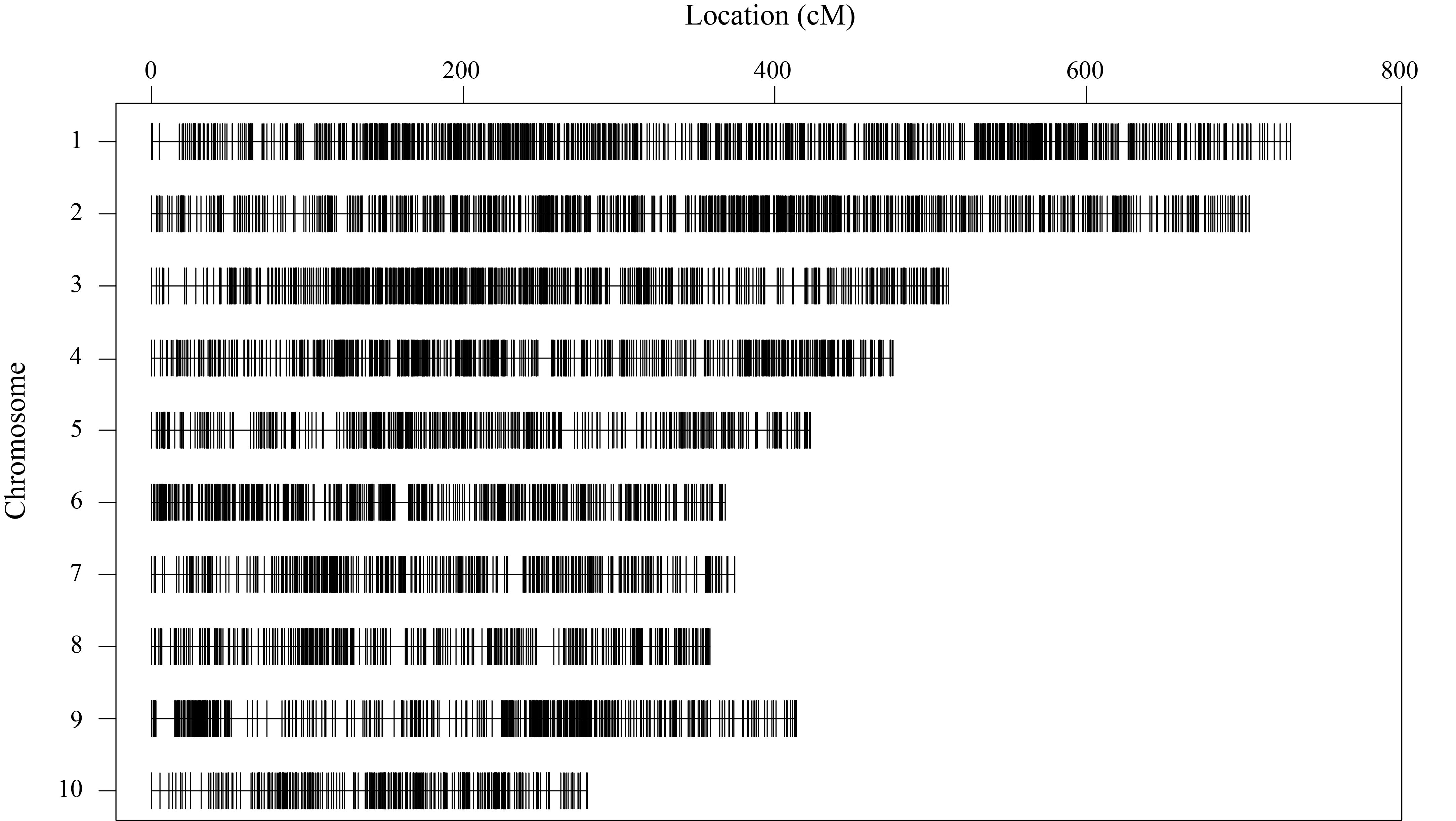

Supplement: Supplementary Figure 1 — Correlation between PH and EH in RIL and two IB populations. The blue bars represent the corresponding phenotype distribution, the red curve is the phenotype distribution curve, and the green curve is the correlation fitting curve for the two phenotype data. *** indicates p<0.001, ** indicates p<0.01, and * indicates p<0.05. [file Image_1.tif]

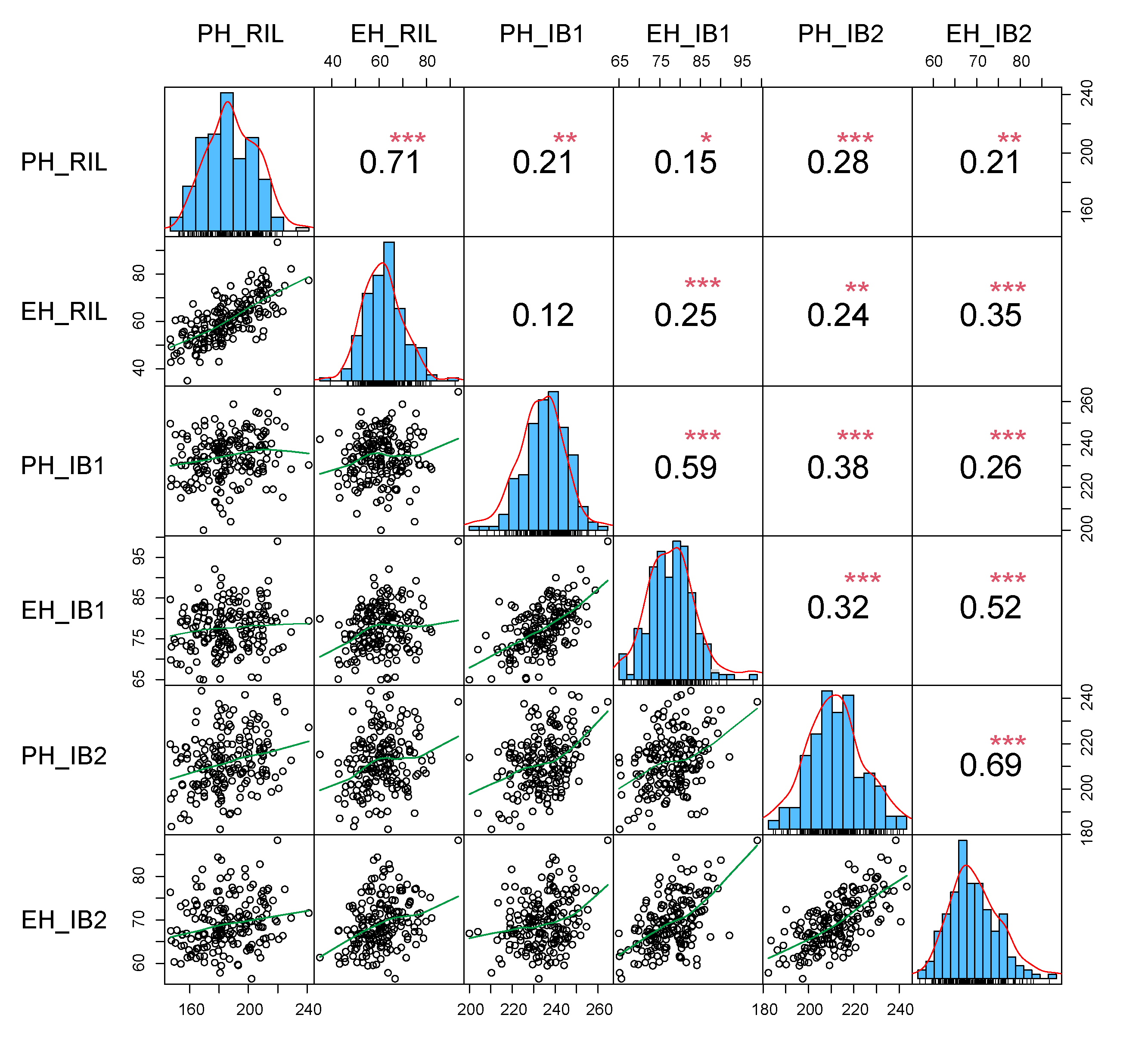

Supplement: Supplementary Figure 2 — Genetic linkage map marker density. [file Image_2.tif]
